# Survival and selection biases in early animal evolution and a source of systematic overestimation in molecular clocks

**DOI:** 10.1098/rsfs.2019.0110

**Published:** 2020-06-12

**Authors:** Graham E. Budd, Richard P. Mann

**Affiliations:** 1 Department of Earth Sciences, Palaeobiology, Uppsala University, Villavägen 16, Uppsala 752 36, Sweden; 2 Department of Statistics, School of Mathematics, University of Leeds, Leeds LS2 9JT, UK; 3 The Alan Turing Institute, London NW1 2DB, UK

**Keywords:** crown groups, diversification rates, the push of the past, molecular clocks, Cambrian explosion

## Abstract

Important evolutionary events such as the Cambrian Explosion have inspired many attempts at explanation: why do they happen when they do? What shapes them, and why do they eventually come to an end? However, much less attention has been paid to the idea of a ‘null hypothesis’—that certain features of such diversifications arise simply through their statistical structure. Such statistical features also appear to influence our perception of the timing of these events. Here, we show in particular that study of unusually large clades leads to systematic overestimates of clade ages from some types of molecular clocks, and that the size of this effect may be enough to account for the puzzling mismatches seen between these molecular clocks and the fossil record. Our analysis of the fossil record of the late Ediacaran to Cambrian suggests that it is likely to be recording a true evolutionary radiation of the bilaterians at this time, and that explanations involving various sorts of cryptic origins for the bilaterians do not seem to be necessary.

## 1. Introduction

The early evolution of the animals remains a remarkably contentious topic, with a lack of agreement even on fundamental issues such as when it took place. The majority view in the field is probably that animals evolved considerably before their undoubted fossil record commenced (e.g. [1–6]). Such a view is based on several lines of evidence: (i) molecular dates, that have consistently placed the timing of (for example) bilaterian origins tens to hundreds of millions of years before the Cambrian [2,4]; (ii) biomarkers that have been used to place the origin of sponges in the Cryogenian [7]; (iii) a general view about timing involving a necessary period of evolution being required before the fossil record (and its biogeography) can be generated [8,9]; and (iv) a view that the fossil record is in general patchy and unlikely to be reliable in any useful way for documenting the precise timing of animal origins (see discussion in [5]).

Standing against this sort of view is the probably minority one that the fossil record of early animals can be read more or less ‘as is’, despite its obvious imperfections [10–13]. In particular, the origin of bilaterian-like trace fossils from later than approximately 558 Ma in the late Ediacaran [14] is seen as an important marker that provides a backstop for the latest time of origin of crown-group bilaterians, and which probably indicates the time of entry of stem-group bilaterians into the fossil record. The rapid, but resolvable, appearance of body and trace fossil taxa in the succeeding Terreneuvian Series is thus seen as the unfolding of the crown group bilaterian radiation, with which this paper is largely concerned. Here then, we will argue for this being the better attested view of the Cambrian explosion, and show one reason why molecular clocks in particular might be poorly estimating it.

**Figure 1.**
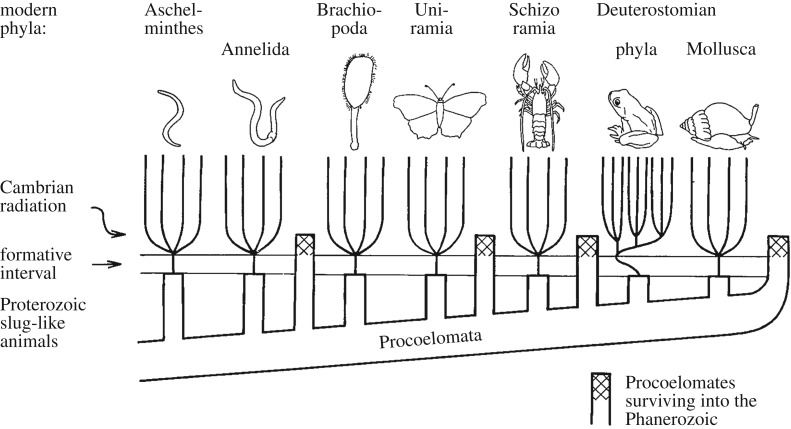
A classical image of the parallel emergence of the phyla around the time of the early Cambrian from relatively simple (and thus unfossilizable) ancestors. Reprinted with permission from Bergström [15].

It seems remarkable, on the face of it, that so well a documented phenomenon as the Cambrian explosion can be open to such divergent interpretations. One reason why this might be the case is that the basic ‘ground rules’ are different in each sort of view. In particular, there is a venerable tradition of viewing the animal phyla as being distinct entities that cannot be easily compared to each other. Thus, it has become quite commonplace to argue that their morphological origins are essentially independent from each other. Whilst such a view has been articulated in various ways by many people, one classical image is the striking one of Bergström 1989 (figure 1; [15]) that shows a series of phyla emerging from a small, slug-like organism (a ‘procoelomate’) during a ‘formative interval’ (cf. Valentine’s ‘roundish flatworm’ [16] and the ‘small, thin’ ancestors of Sperling & Stockey [4]). A more extreme version of such ideas is that the earliest bilaterians closely resembled the planktonic larvae of the extant clades (e.g. [17], drawing on the tradition that can be ultimately traced to Haeckel [18]). Buttressed by various geological and developmental arguments, this hypothesis posits that such small, and by their very nature hard-to-preserve tiny animals are meant to persist up many, if not all stem groups that lead to the crown-group bilaterian phyla. Hence, the appearance of undoubted bilaterian fossils represents in each case the transition from the unfossilizable proto-phylum member to a full-blown fossilizable taxon. Such a transition could be triggered either by an environmental stimulus (typically oxygen levels rising) or presumably by some necessary level of ecological complexity being reached.

It is surprisingly difficult to unpack the entire set of assumptions and traditions that lie behind the first of these views, but some features stand out and can be critically examined. The first is the assumption that for all or at least many bilaterian clades, the ancestral state was a small body size which would be difficult to record in the fossil record [19]. Small organisms often lack key features such as complex musculature, body cavities and appendages, and it seems that the critical body length for these purposes is around 1 mm (see discussion in [12]). Many living protostomes *are* indeed tiny, and some phylogenetic reconstructions have suggested this is ancestral for the clade as a whole [20]. Without a reliable and fully resolved protostome phylogeny, such a view is difficult to assess. Nevertheless, in recent years some relatively large Cambrian members of clades that today consist of meiofaunal organisms have been discovered (e.g. [21–24]). Of course, simply finding a single large member of a clade is not equivalent to demonstrating that large body size is the ancestral state, but it does weaken the assumption that small body size *must* be, especially given evidence that old fossils preserve more plesiomorphic character states than recent taxa do (having had less time to shed them; [25]; cf. [26]).

Another point of dispute has been the placement of the ‘Xenacoelomorpha’, a clade that consists of *Xenoturbella* and acoel and nematodermatid flatworms; different analyses have placed them either as the sister group to all other bilaterians [27,28], or as sister group to the Ambulacraria (echinoderms and hemichordates) [29]. Such a clade, and others such as the platyhelminths, would naturally be extremely hard to preserve in the fossil record, and if all animals were like this, then one should expect enormous mismatches between the true time of origin of a particular clade and its entry in the record, if this happened at all. It should be stressed, however, that even if such a clade *is* the sister group to all other bilaterians, it does not immediately follow that its simple morphology must represent the ancestral condition for bilaterians as a whole (as in [27]). Determining this would require detailed character reconstruction at the base of the *rest* of the bilaterians (which is currently a matter of considerable dispute) and reference to the cnidarian outgroup. It is thus not easy to rule out the possibility that stem-group Xenacoelomorpha members were at some point relatively large worm-like organisms (cf. [30]).

Body size is by no means the only marker for preservability, and perhaps not even a very good one. However, the trace fossil record *is* one that is tied to body size, and it is difficult to imagine a high abundance of relatively large bilaterians existing without any sort of perceivable trace fossil record. Furthermore, even if an ancestral small organism (or one that could not leave trace fossils) was inferred (the broad conclusions of several papers suggesting extreme convergence between protostomes and deuterostomes based on molecular development such as [31]), it would be required to stay in this state without any ecological innovation for many millions of years. Trace fossils from the terminal Ediacaran can be quite large [32], and—if we accept that they represent stem-group, or even early crown-group bilaterians—it follows that some of these were of large size too. If *these* bilaterians could be large at this time, why not the others implied by molecular clock analyses, unless they were yet to evolve?

If we accept that ancestral bilaterians *were* small, the implication for the fossil record would be that as the stem-group members of many phyla crossed into the Cambrian, they would simultaneously each have to develop into large animals, in a highly unparsimonious way (but see [33] for a discussion of a potential analogy in mammalian evolution). Finally, it should be noted that at least putative trace fossils of meiofaunal organisms have been reported from Brazil [34], suggesting (if correctly assigned) that even tiny organisms might leave traces. The trace fossil nature of these has, however, been questioned [35].

The problem of suggesting multiple attainments of large and fossilizable body size as a solution to the problem of the lack of Ediacaran crown group bilaterians is partly concealed by the low sampling of present day diversity in summary cladograms, because phyla are often (and perforce) represented by a single lineage (see e.g. [5]). However, if the crown group of any particular clade of large animals is thought to have emerged before the Cambrian, then *all* crown group lineages of that phylum crossing the boundary would themselves have to independently develop large (and complex) organization. This itself is striking: why would it be the case that of all the implied crown group bilaterian diversity in the Precambrian, no lineage ever hit upon a way of attaining large body size? Such arguments have recently gained new potency because of the growing and widespread recognition that the large Ediacaran organisms indeed lie within stem groups to various animal groups (cnidarians, ctenophores, bilaterians, etc. [36–39]). If, then, the presence of various large metazoans that are not crown group bilaterians (complete with their at least putative trace fossils (e.g. [40,41])) is acknowledged during the Ediacaran, why are there no crown-group bilaterians? And if crown group bilaterians really emerged at *ca* 630–600 Ma, why do *stem group* forms only apparently emerge *after* this time? We believe that the combination of these factors makes the presence of bilaterians deep in the Ediacaran or even earlier very unlikely indeed.

**Figure 2.**
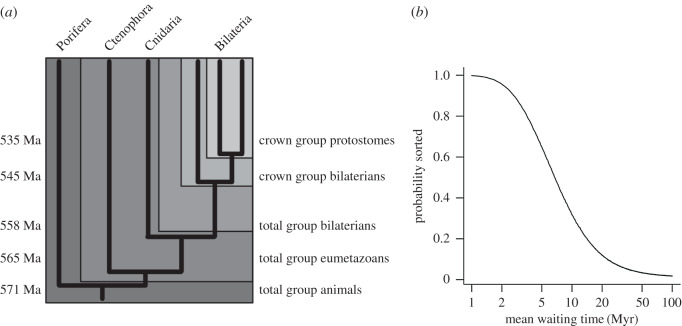
Using the resolution of the Ediacaran–Cambrian fossil record to estimate the gap between the true and fossil appearance of clades. The fossil record is used to identify the first appearance of five nested clades, the appearance of one of which logically implies the earlier appearance of all the more inclusive ones. We model the time of appearance of each clade in the fossil record after its true origin with an exponential distribution with varying waiting times (*x* axis), and ask what proportion of 100 000 simulations reproduced the appearance in the correct order. Note that if the waiting time is more than about 10 Myr, the observed pattern rapidly becomes highly unlikely. Data: Total group animals: Drook Formation, Newfoundland, 571 Ma [50]. Various frondose taxa [51] that have been broadly suggested to cluster around the base of total group, or into the crown group of animals [36,37]. Total group Eumetazoa: Mistaken Point Formation. Newfoundland, 566 Ma [50]. Metazoan-grade trace fossils suggesting a muscular organism [52]. Total group Bilateria: Ust-Pinega Formation Sequence A, Zimnie Gory section, White Sea, *ca* 556 Ma [14]. The oldest bilaterian-aspect trace fossils are from below an unconformity directly below an ash bed dated to *ca* 555 Ma. Although their maximum age is not constrained, it is probably within a few million years of 555 Ma. Crown group Bilateria: Urusis Formation, Namibia, *ca* 546 Ma [53]. *Treptichnus* sp. burrow systems. Recent work suggesting that scalidophorans may have been responsible for Cambrian treptichnids (e.g. [54]) implies that these early treptichnids may be made by organisms close to or within the bilaterian crown group. They lie below an ash bed dated to 545 Ma, and above an unconformity above rocks dated to 548 Ma. Crown group Protostomia: e.g. Ust’-Yudoma Formation, Siberia, *ca* 535 Ma ([45], see their supplementary information for details; for dating see e.g. [44]). Protoconodonts (assignable to total-group Chaetognatha) such as *Protohertzina anabarica* are often considered slightly to predate other obvious protostomes in the small skeletal fauna.

Such a view receives substantial support from birth–death modelling [42,43], which suggests that crown groups emerge rapidly and swamp their stem groups in short order. In such a view, if the crown-group bilaterians emerged during the early Ediacaran (cf. [2]), then modelling would suggest that by the time of the opening of the Cambrian, there should be many thousands of species, diversified into many different crown group lineages. Yet, somehow, not a single body fossil of an indisputable crown-group bilaterian has ever been found before the Cambrian. Just as strikingly, when animals *do* appear in the fossil record in the Cambrian, the fossil record develops in a way that makes it look as if a real diversification is going on (see e.g. [10,44–48]; a pattern that is consonant across the small skeletal, carbonaceous and trace fossil records [47]). To wit: at around 571 Ma or so appear fossils that may be assigned to stem or early crown group animals [36,37,49–51]; at around 566 Ma comes the first evidence of eumetazoan-grade organisms in the form of trace fossils [52]; no later than around 555 Ma are found complex (i.e. bilaterian) trace fossils and taxa assignable to stem cnidarian, ctenophore and bilaterian grades [14,36]; by around 545 Ma near the end of the Ediacaran comes a diversification of large trace fossils (assignable to late stem and perhaps early crown group bilaterians [53]); and rather early in the Cambrian, perhaps 535 Ma come the first definitive crown group bilaterians (e.g. protoconodonts [45]), followed by lophotrochozoan conchs, total group arthropod traces, etc. Thus, we have an appearance in the fossil record of five grades of organization (total-group animal; total-group eumetazoan; total-group bilaterian; crown-group bilaterian; and crown-group subclade of bilaterians) with approximately 10 Myr in between each, that appear in the right order. This pattern would be very unlikely if different grades of animal evolved some time in the distant past and then randomly appeared in the fossil record; furthermore, similar to the Rayleigh Criterion in optics, it suggests that the uncertainty around the true time of appearance is not likely to exceed the size of the gaps—i.e. these times of entry are unlikely to be more than 10 Myr after the true origin of the groups in question (figure 2). Such a pattern has also been noted in the unfolding of the early angiosperm fossil record [55], despite molecular clock evidence for a deeper origin [56,57].

No satisfactory explanation of such patterns has been undertaken if the whole clade really diversified much earlier. In the rest of this contribution, then, we take as our view that crown-group bilaterians emerged late (probably just before the beginning of the Cambrian [58]). We wish to examine two features of this emergence, both through birth–death modelling: (i) why do body plan features appear to emerge so rapidly in the Cambrian, and (ii) why might molecular clocks be overestimating the timing of the origins of clades?

## 2. Birth–death models and their biases

**Figure 3.**
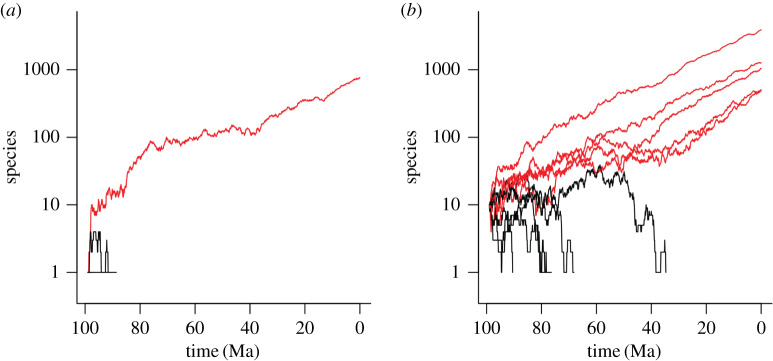
Two ‘gambler’s ruin’ simulations with an exponential birth–death process, with a speciation rate of 0.544 Myr^−1^ and an extinction rate of 0.5 Myr^−1^. (*a*) Starting with one species; (*b*) starting with 10 species. Red lines are successful clades that manage to escape from the absorbing boundary of extinction; black ones, clades that go extinct. In the case of initially small clades (*a*), most rapidly go extinct without reaching a substantial size. Initially larger clades (*b*) more often survive; note that those that go extinct tend to behave like the survivors until they rather rapidly collapse to extinction. R code used for generating the figures is included in the electronic supplementary material.

Patterns of diversification have been long studied through birth–death models, with important early papers being [59,60]; with very significant early contributions by Raup, whose 1983 paper [61] on the early origins of major groups is an underappreciated classic (for an update of this work, and a general discussion of the fossil record and birth–death models, see [43]). Recent important developments in this general area include e.g. [62,63]. Simple homogeneous birth–death models assign constant rates of extinction and speciation to a group; an end member is the so-called Yule process that has only speciation and no extinction [64]. However, despite the presence of these constants in the models, a wide range of stochastic outcomes can emerge from them (compare: even if one has a constant 1 in 6 chance of rolling a 6 with an unbiased die, the number of sixes actually rolled in a trial set of 20 rolls will vary greatly from one trial to the next). In particular, if there is any sort of significant extinction rate, then clades are logically unlikely to survive for long. Clades that do survive to the present from a distant time in the past (as Cambrian clades would represent) thus represent a small subset of all possible clades. Such survival can be compared to a classical ‘gambler’s ruin’ process (cf. page 45 ff. of ref. [65]), with the critical difference being that in an exponential process (as opposed to the simple random walk of Raup), it is possible (rarely) to escape from the absorbing boundary that extinction represents (figure 3).

Such survivors thus represent a very biased subset of all possible outcomes of the birth–death process, and the most important component of their peculiar characteristics was briefly identified by Nee *et al.* [59] as the ‘push of the past’. Essentially, the push of the past is the chance higher-than-normal rate of diversification present in the early species of a successful clade. As discussed by Budd & Mann [43], this early burst of diversification can only be seen by considering the observed rate in species as a whole (i.e. including both extinct species and lineages leading to modern groups).

The push of the past has been almost entirely ignored, because most phylogenetic work involving birth–death models has involved the modelling of the so-called reconstructed evolutionary process. This essentially involves the modelling of the ‘lineages’ that give rise to modern day diversity, and which can be reconstructed backwards in time through the coalescence process (e.g. [66]). Such lineages are, in short, those represented by the terminal and internal branches of a phylogeny. Rather surprisingly these lineages, although speciating twice as fast as the background rate [43,66], show little systematic variation in the rate in which they are created along them until the present is nearly reached, and thus show no ‘push of the past’ effect, at least as defined by [43,59], even though early diversification rate changes of lineages are sometimes mistakenly referred to as such (e.g. [67]). Early high rates of *lineage* creation, then, must rely on a different feature.

**Figure 4.**
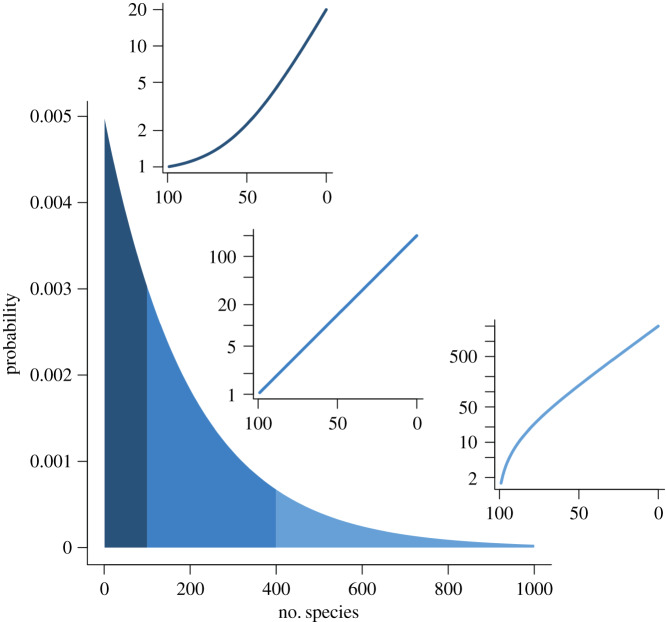
The geometric probability distribution of Yule-process generated crown group sizes for crown groups of age 100 Ma, mean size of 200 and birth rate of 0.0530. The distribution is broadly divided into small (left), medium (middle) and large (right) clades. The middle section runs from clades that are half (100) to twice (400) the mean size of 200 species. Representative lineage-through-time (LTT) plots are provided for each sector (compare figure 7) with retrospective time in million years on the *x* axis and the number of lineages of the *y* axis. The small clade representative LTT is 10 times too small, and the large clade representative LTT 10 times too large, compared to the mean.

Various explanations have been posited for observed instances of such early bursts of lineage diversification [68–71], and the general pattern has been referred to as the ‘large clade effect’ (LCE [43]; for a mathematical characterization, see [42]). Whatever their underlying causes, early bursts can also be seen as features of the general stochasticity of birth–death processes ([43]; cf. [72]). Nevertheless, this is not a *survivorship* bias, because all clades that have lineages must tautologically have survived to the present day. However, survivorship could occur by only one lineage of a particular clade living in the present day, or by many thousands, even with the same diversification parameters. If one examines only the *large* examples out of these many possibilities, it will be seen that they will generally have created a larger-than-expected number of lineages early on in their history, giving a characteristic ‘bulge’ in the lineage-through-time (LTT) plot of the clade (figure 4; cf. fig. 7 of ref. [43]). This, then, is a *selection*, not *survivorship* bias of the process (cf. [73], who discusses the perils of treating large clades as representative of the evolutionary process as a whole).

A few papers have explicitly examined these early diversification bursts considered as features of unusually large clades (e.g. [43,72,74]). However, they are normally subsumed under a different sort of category: ‘diversity-dependent’ diversification (DDD). Such models make the assumption that as a characteristic carrying capacity of a particular clade is reached, the rate of diversification will asymptotically slow down, until a steady state is reached. Although we are rather sceptical about the biological reality of the ecological underpinnings of such models, we note that in any case the typical patterns of the LCE and DDD closely resemble each other, and it is not clear that the two can be easily distinguished [42,43].

Two sorts of more or less distinct bias can thus be distinguished in birth–death modelling of the evolutionary process: the push of the past *survivorship* bias of the process as a whole, and the large clade effect *selection* bias that further emerges by only examining unusually large living clades. We now wish to explore some of the features that these biases might introduce into patterns of evolution, in particular the rapid formation of body plans and how they might affect molecular clocks.

## 3. Early bursts of molecular and morphological evolution: the case of the arthropods

In order to introduce the rates of morphological (and, indeed, molecular) evolution into discussions about trees based on birth–death models, it is probably necessary to make a further assumption, i.e. that there is some sort of relationship between rates of speciation and rates of molecular and/or morphological change. Such a relationship seems intuitive, because eventually all species must be distinguished by certain morphological and molecular synapomorphies, but the empirical evidence for this has been mixed (see e.g. [75]). One potential reason for this is that molecular phylogenies are perforce based on the reconstructed evolutionary process which cannot include extinctions within it (lineages, by definition, cannot include extinction). Any examination of rates of molecular or phenotypic change in such trees can thus only examine the relationship between such rates and the rate of *lineage production*; and the latter will bear little resemblance to the rate of true *speciation* that includes all extinct taxa too. Only if extinction rates are very low will there be a reasonably close relationship between the two, because then any speciation event in the past will have a good chance of giving rise to a lineage.

**Figure 5.**
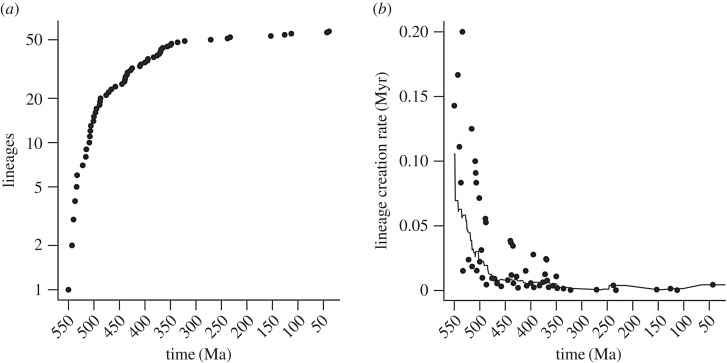
(*a*) The ‘lineage-through-time’ (LTT) plot reconstructed from the data of Lee *et al.* [76]. Note, owing to (inevitable) undersampling, that the LTT plot flattens out towards the recent, but that it starts with a pronounced bulge characteristic of large clades. (*b*) The reconstructed rate of lineage production through time. Each point is the inverse of total branch length between successive lineage creations; a 50 Myr smoothed average line is also included. This effect with that rate of slow-down of molecular and morphological rates of change in Lee *et al.* [76], fig. 3.

We explore some of the problems discussed above by examining the important study of rates of evolution of arthropods by Lee *et al.* [76] using the phylogenetic reconstruction software BEAST, which they take as a proxy for the Cambrian explosion as a whole. They showed that if the time of origin of the arthropods was fixed by the fossil record at around 555 Ma, then arthropods evolved very rapidly during the Cambrian, with initially high rates of both molecular and morphological change across the early lineages. Arthropods, above all other clades, are likely to be a ‘large clade’ in the sense of [43], given that they are much larger than any other clade of a comparable age that does not include them. This view is supported by the LTT plot that can be reconstructed from the data of Lee *et al.* [76] (figure 5), showing a large early bulge. In addition, because of the inevitably very low sampling rate (*ca* 50 taxa across the probable millions of extant species), the LTT shows a rapid flattening out as the present is approached (i.e. given true present day diversity, one would expect many more lineages actually to have been created as the present day is approached). We note that there is a range of possibilities within any diversification (note error limits in fig. 7 of [43]). The arthropods indeed may be a case in point, as the huge insect diversification takes place some time after the origin of the clade. How such heterogeneities affect the behaviour of a reconstructed clade remains to be investigated.

A notable feature of the data provided by Lee *et al.* [76] is that when one compares the rate of lineage creation to the rates of molecular/morphological evolution, they counterbalance each other, so that the mean number of molecular (or morphological) changes per lineage creation remains constant through time. Thus, the curve showing the rate of decline of lineage creation (figure 5*b*) is very similar to their curves showing the rate of decline of morphological/molecular change (their fig. 3). Furthermore, they show that the size of the initial burst of evolution is dependent on where the root of tree is placed; if deeper, the initial rates are lower. These patterns imply that lineage creation rate (as opposed to speciation rate) and evolution rates are not independent (cf. [77,78]). Why might the creation of a lineage be accompanied a fixed amount of molecular and morphological change?

If one makes the assumption that rates of morphological/molecular change correlate in some sense with rates of speciation (see above and discussion in [43]), it follows that the large clade effect would not predict such a slowing down of rates along the lineages, which only have a slightly elevated initial speciation rate in large clades compared to normal ones [43]. Several other possibilities present themselves, however. The first is that the root of the arthropods is really much deeper than the fossil record suggests (but see below), and that artificially compressing their early evolution has the side effect of increasing early rates of evolution too. The second is that there really is a large clade effect (i.e. early arthropod lineages do appear much faster than expected), and that either the algorithms used for distributing character change across the tree do not take this into account, or the amount of data is insufficient to probe the processes very deep in the tree (see sensitivity analysis below). Finally, there is the possibility that there is an as yet poorly understood lower-level process (involving ecology, competition etc.) that really does connect rate of lineage creation with that of underlying evolutionary change. Despite the difficulties of understanding what happens along the lineages of a clade, it nevertheless remains likely that successful clades will show high rates of evolutionary change in their early history when averaged across both plesions and lineages, because of the overall push of the past effect [43], but concentrated into the early lineage(s). Such rapid early evolution will however, be a necessary feature of the emergence of a successful clade, and not its cause, as in the ‘key innovation’ concept (see discussion in e.g. [79]). One further selection bias may enter here. Phyla are commonly thought of as (often) large clades that are morphologically distinct from each other. Unusually large clades should have short stem groups [42,43], and all things being equal this should imply a relatively short period of time for evolutionary change to accumulate; yet the distinctness criterion for phyla suggests the opposite, since the accumulation of distinctiveness occurs by extinction in the stem group [10,80] and therefore selecting phyla through distinctiveness selects in favour of longer stem groups. Although the overall effect of such a bias is uncertain, it should probably be considered as an important factor when trying to understand the disparity differences between phyla when considered as distinct evolutionary units (e.g. [80]).

## 4. Molecular clocks and the large clade effect

Because almost all molecular data are from living organisms, it follows that they bear largely upon the reconstructed evolutionary process. A further consequence is thus that molecular data are in principle not affected by changes in rates of speciation, which are largely constant along the lineages, even in large clades (until the recent is approached at least [43]). However, we have previously suggested that molecular reconstruction might nevertheless be more subtly affected by the large clade effect [43]. Whilst this influence is potentially wide-ranging, here we focus on one part of it, which is the influence of the LCE on molecular clock estimates. We emphasize that in this section, we take a rarefied approach to the topic to isolate the potential influence of the large clade effect, without attempting to reproduce every aspect of how molecular clocks are typically constructed and calibrated.

Modern-day molecular clocks rely on three components: (i) a model of molecular evolution; (ii) a model of tree structure; and (iii) a model of the distribution of clock rates across the tree [81,82]. It is possible to date nodes on a fixed tree topology, such as in MCMCTree. Conversely, other software such as e.g. BEAST and BEAST2 also allow you to simultaneously estimate a phylogenetic reconstruction, branching model and rate of substitution along the lineages, often employing so-called ‘relaxed clock’ methods (e.g. [83,84]). As far as this paper is concerned, the major difference between the two methods is how the tree model is integrated with calibration information about the ages of particular nodes (see [85–90]). In MCMCTree, the so-called ‘conditional prior’ is used, which broadly constructs a time prior based on specified calibrated distributions of the calibrated nodes, and then conditions a birth–death process on this to create a prior for the uncalibrated nodes. Conversely, in the so-called ‘multiplicative prior’ that was originally implemented in BEAST and BEAST2, a birth–death time prior is created for all the nodes, and then multiplied by the time prior over the calibrated nodes. As has been pointed out several times, such a method is formally incoherent, and also potentially creates some unusual artefacts in the overall prior [87]. However, it has the advantage of considering all the nodes as part of a single process. The conditional prior, on the other hand, does not incorporate information from the birth–death prior into the relationship between the calibrated nodes (which necessarily includes the basal node in MCMCTree). These two broad methods of creating the time prior do appear to lead to differing results, especially for the root [56,89].

It is rather noticeable that in many cases, molecular clocks considerably overestimate times of clade origins when measured against the fossil record, and the origin of the bilaterians has been one of several classical instances of this pattern (for the case of angiosperms, see e.g. [55] and the particularly trenchant discussion by [91]). Often such mismatches arise through rather imprecise or inadequate calibration of clocks [92,93], and indeed better attention to calibration has certainly narrowed the fossil–molecular clock gap in many instances (see e.g. [94]). Even so, despite rather close interrogation, molecular clock estimates of bilaterian origins, although exhibiting a considerable range of possible dates, still firmly place them deep in the Ediacaran or even earlier, even when the uncertainty limits on the estimates are taken into account [2,5]. This is also seen, at least by implication, in the BEAST analysis of [82].

Many possible reasons for problems with molecular clocks have been discussed, including those concerning heterogeneous molecular evolution [95], date calibration [92,96] and various issues of sampling and tree priors [97–100]. Without denying any of these issues, many of which can and have been accounted for, we wish here to take a somewhat broader perspective of the relationship between tree model and molecular clock results. Given our reasoning above, we are led to ask the question: if the chronology of bilaterian/animal evolution that we have reasoned from the fossil record is accepted, why might molecular clocks instead be systematically inaccurate? This is opposite to the traditional perspective of examining ways in which the fossil record might be misleading in light of the acceptance of molecular timescales. Here, then, we wish comment on two topics in particular: the role of unusual clade size in molecular clock bias, and the oft-discussed role of node calibration.

### 4.1. Lineage-through-time plots and times of origins: general considerations

**Figure 6.**
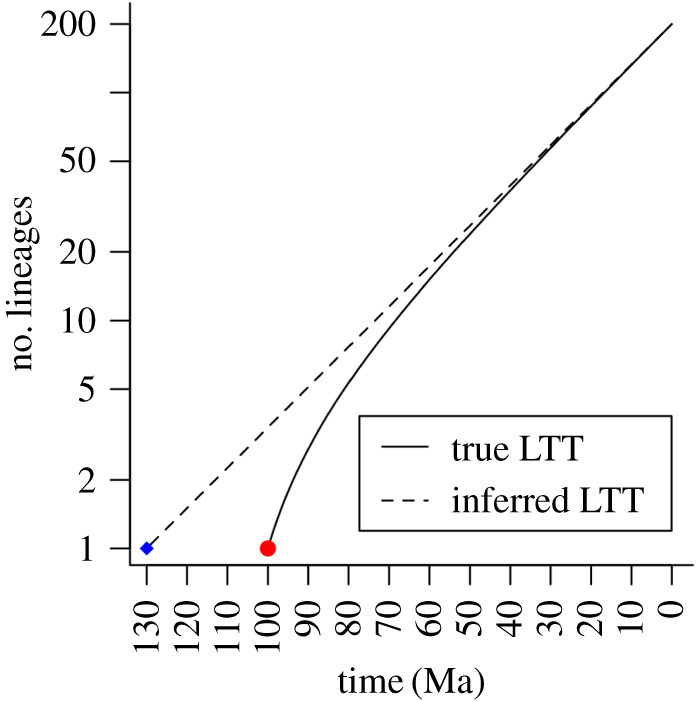
The possibilities of poor inference from unusually large clades. The solid line represents the true LTT plot for an usually large clade that would normally have been expected to produce *ca* 60 species in the present after diversifying for 100 Ma (diversification rate 0.0407), but in fact produced 200. Extrapolating the asymptotic slope of the curve at the recent backwards assuming the clade was a normal size (dashed line ending in blue dot) would imply an origin at 130 Ma. For simplicity, we have chosen a Yule process, but a similar principle would hold for a birth–death model too.

By reconstructing a tree, and with some dating constraints on it (by estimating some combination of the rate of molecular change, ages of tips or of nodes), the lineage-through-time (LTT) plot should be expected to yield an estimate of the base of the clade (figure 6). For normally sized clades, the LTT plot (when not close to the present) is a straight line on a semi-log plot, with slope *λ* − *μ* (where *λ* is the unit rate of speciation and *μ* the unit rate of extinction). However, in an unusually large clade, the early part of the LTT shows a much higher slope than that of the central part (which remains of slope *λ* − *μ*), implying the lineages accumulate faster than one would expect. Extrapolation from the central part of the plot might thus be expected to yield an erroneously deeper origin of the clade (figure 6). In the case of the Yule process, this is a straightforward calculation as the expected time to produce a clade of *N* species is ln(*N*)/*λ* and to produce a clade of *αN* species is ln(*αN*)/*λ*; the expected time difference is thus ln(*α*)/*λ*.

Bayesian phylogenetic inference packages such as BEAST and BEAST2 [101] can employ a birth–death (or Yule) model for their clade inference (it has recently been shown, however, that initial model selection has little effect on the results obtained from BEAST2 at least [102]; although the exceptions are of interest). Such inference discovers the most (a posteriori) likely trees for any given set of input data. A priori, this will favour trees with an early straight-line LTT, because this is the central outcome of the birth–death (or Yule) process, and in BEAST2, the birth–death prior can be applied to all the nodes (see discussion below). If the true process led, however, to an unusually large (or small) clade, then the molecular data may be in tension with this central expectation; in such trees, the early LTT plot is either curved up or is flatter than expected, respectively. In other words, the shape of the tree, and the rate of accumulation of change upon it, will be telling different stories. In such a scenario (we predict), the inferred time of origin of the clade will emerge as a compromise between the two, with the exact balance being determined by the relative weight given to each and the relative flexibility of each prior. Even if the time of origin of a clade is well known, and its number of species in the present, the true extent of the LCE might not be inferred, because it is possible to reconstruct the generating process by a higher background diversification rate.

### 4.2. BEAST2 recovery of simulated data

We have chosen to examine the influence of the LCE on molecular clocks estimated by BEAST2 by a simulation approach. Using the *TreeSim* package in R [103] we first simulated eight sets of five trees (table 1), each with 200 terminal taxa. Each tree was fixed to a height of 100 Myr (to the most recent common ancestor), that is, from the beginning of the crown group (i.e. from two founding species, not one as would be the case for simulating from the base of the total group. Note this implies a two-trial negative binomial rather than geometric distribution for the probability of obtaining the 200 taxa. The diversification rate in each set was chosen so that the actual 200 taxa in each of the sets of five trees represented 0.1, 0.2, 0.5, 1, 2, 3, 5 and 10 times the number that would be expected given the diversification parameters chosen. For further simplicity, a Yule process (i.e. with no extinction) was chosen for the tree generation. After generating these trees we further simulated a process of molecular evolution along the branches from the most recent common ancestor to the terminal taxa. For each tree we simulated 1000 base pair sites, with a homogeneous Poisson process for nucleotide substitutions, utilizing a substitution rate of 0.03 per site per million years (i.e. 0.01 per possible transition), using the *phytools* R package [104]. These alignments were then ported into BeauTi 2.5.2 [101] to generate XML files.

**Table 1.** The parameters of each set of five simulated Yule-process trees (giving 40 simulations in total). Note that trees are simulated from the origin of the crown group (i.e. starting with two lineages).

set no.	no. of taxa	tree height	div. rate (*d*)	expected no. of taxa given *d*
1	200	100	0.0691	2000 (clade 0.1× expected size)
2	200	100	0.0621	1000 (0.2×)
3	200	100	0.0530	400 (0.5×)
4	200	100	0.0461	200 (1×)
5	200	100	0.0391	100 (2×)
6	200	100	0.0351	67 (3×)
7	200	100	0.0300	40 (5×)
8	200	100	0.0230	20 (10×)

For the BEAST2 version 2.5.2 recovery of the simulated data, we set a strict clock rate fixed to the true value of 0.03, a Yule process tree prior (with the default uniform [− ∞, ∞] prior on the birth rate), and a MCMC chain of 20 000 000 samples, reduced to 20 000 trees after thinning and burn-in, for further analysis with Tracer [105]. The R code used for generating trees and their associated molecular data, and the XML files are all included in the electronic supplementary material.

**Figure 7.**
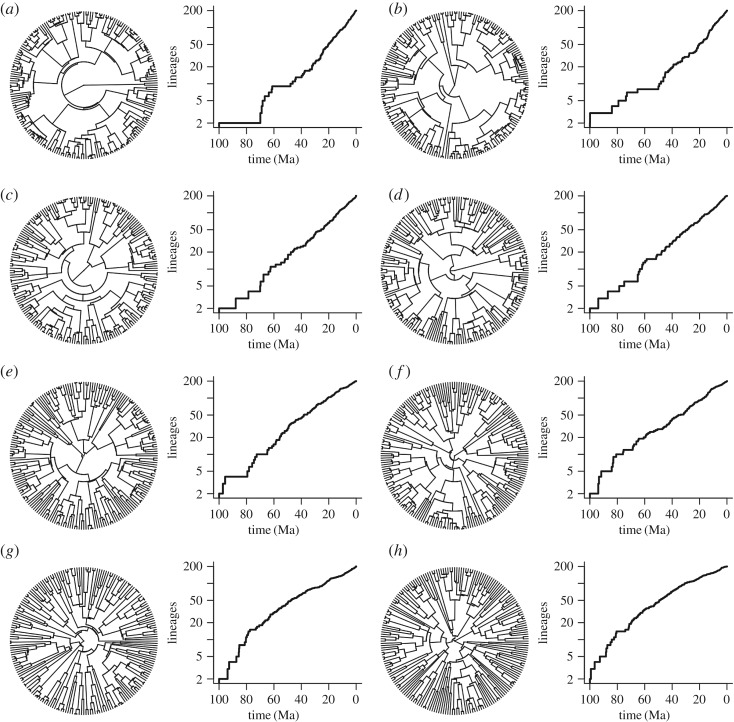
Representative trees and LTT plots for clades that are (*a*) 0.1 (*b*) 0.2 (*c*) 0.5 (*d*) 1 (*e*) 2 (*f* ) 3 (*g*) 5 and (*h*) 10 times too large for their diversification parameters (table 1). Note how the LTT plots move from bulging downwards to bulging upwards. The slope of the LTT when it is distant from its origin in each case is approximated by the relevant diversification rate given in table 1.

For illustrative purposes, we show an exemplar simulated clade of each set, together with its LTT plot (figure 7*d* is the expected clade size for the given diversification rate). Note that in the smaller-than-expected clades (figure 7*a*–*c*), there is a delayed lineage diversification, leading to a depressed early LTT; whereas in the larger-than-expected clades (figure 7*e*–*h*), the opposite pertains: more lineages emerge early on, and there is an increasingly pronounced bulge in the early LTT plot.

### 4.3. Results

**Figure 8.**
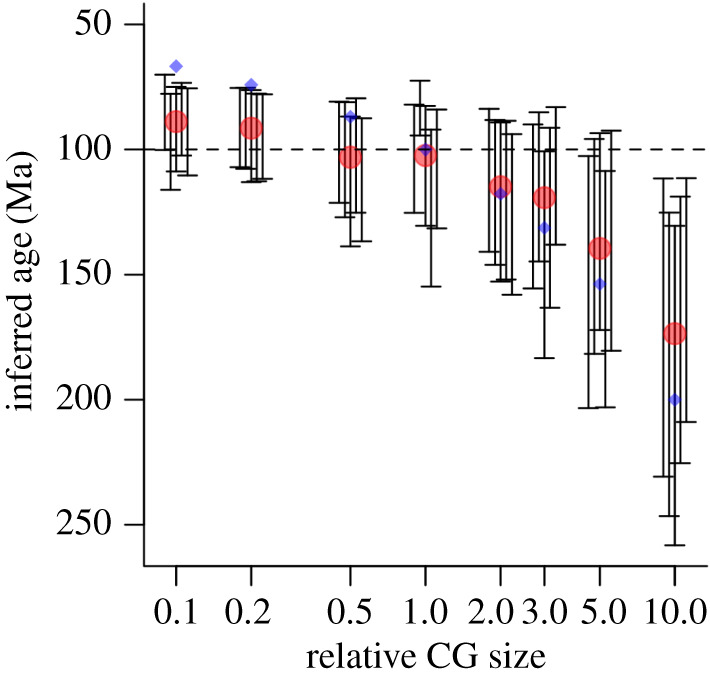
Inference errors of tree height in BEAST2. Eight groups of five inferences corresponding to the diversifications in table 1 are shown as a plot of inferred tree height (i.e crown group age) against relative clade size. The true tree height in each case is 100 Ma (dashed line). The red dots represent the mean of the means of the five runs in each set, and the blue dots represent the extrapolated inferred tree height from figure 6. Note that in each case (except the normal sized clade where all three are close), the red dots lie between the real value and the blue dot. The vertical black lines represent the 95% HPD intervals in each run.

We show the results of inference of tree height on all 40 sets of simulated data in figure 8, grouped by (true) relative clade size. In each set of five, we show the 95% high posterior density (HPD) interval and the mean of the five means. In addition, we plot the naive expected value of the tree height based on figure 6. All the outputs reached a satisfactory value (i.e. >200) of effective sample size (ESS) for all parameters. The most notable result of the plot is that in the large clade cases, the tree height is consistently overestimated, and in the small clade cases, underestimated, with a clear trend of effect between them. In each case, as predicted, the mean of the mean plot falls between the true value of 100 Myr and the height calculated from a naive extrapolation of the recent diversification rate as shown in figure 6. We note two significant sources of stochasticity: that of the individual HPD intervals, and variation between the five simulations of each parameter set that result from the inherent stochasticity of the Yule process. Despite these sources of variability though, the overall trend from larger-than-expected to smaller-than-expected clades is very clear.

### 4.4. Discussion

As predicted, choosing to examine trees with known unusual features exerted pressure on the reconstruction process (cf. the closing comments of [95], who hint at this effect), and the clear outcome was that larger-than-expected clades had their heights overestimated by BEAST2, with smaller-than-expected clades being underestimated. Although the values we chose for our simulations were illustrative, the scale factors are absolute, and the timescale is arbitrary, in the sense that one time unit can represent any amount of real time. It is thus possible to translate our results directly into a timescale that would be comparable to that of the Cambrian explosion. For example, for a clade that had a crown group two times too big, and with a real origin at *ca* 545 Ma, our results suggest that an origin at *ca* 625 Ma would be inferred. It should be reiterated however that the actual value will depend on the quantity and quality of the data, both of calibration and sequence data. Our results here nevertheless serve to show that the scale of overshoot in empirical studies is consistent with our simulated results. This result is significant when compared to BEAST estimates of the rate of the Cambrian explosion (e.g. [82] which imply a deep root for both the arthropods and animals more generally, unless a very strict young root is imposed.

**Figure 9.**
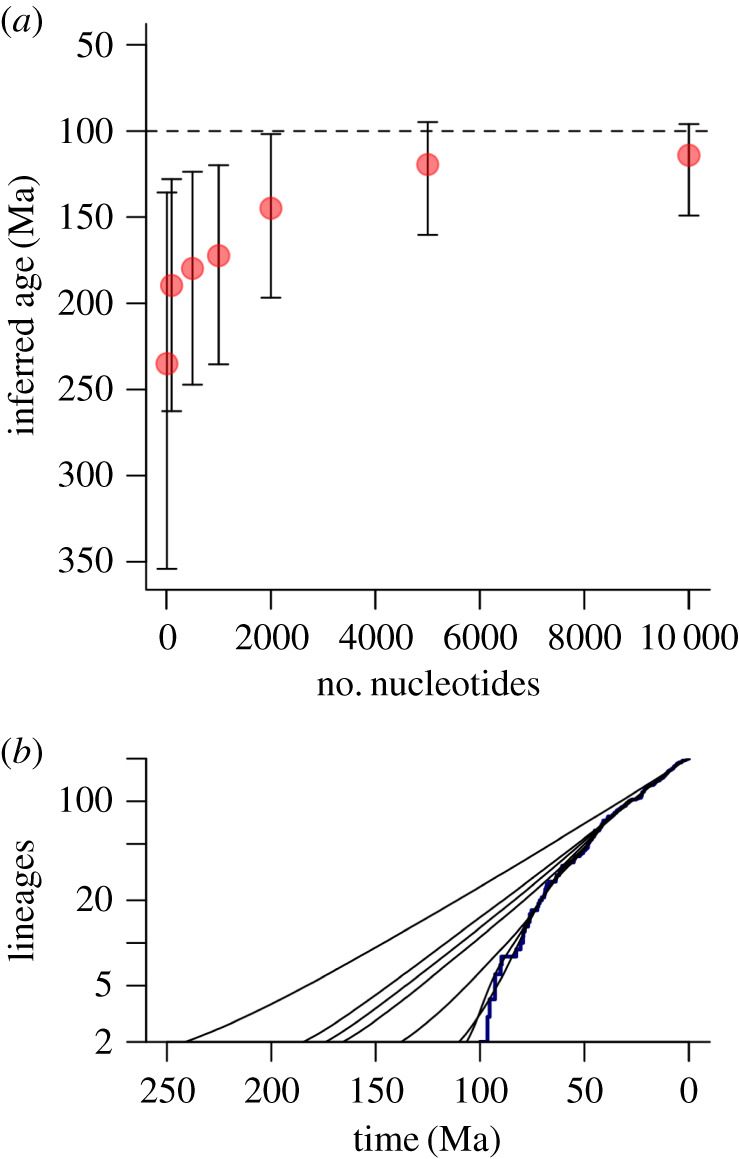
Sensitivity of the large clade effect to amount of data in our simulations. (*a*) For a 10 times too large crown clade, the inferred age for 10, 100, 500, 1000, 2000, 5000 and 10 000 nucleotide base pairs are shown, showing an asymptotic effect. Dashed line shows the true crown group age of 100 Ma. (*b*) Reconstructed LTT plots for the same dataset, showing that as the dataset increases, the true LTT (here in bold) becomes better approximated.

For any given birth–death or Yule diversification process, there is, from figure 4, a geometric distribution for the size of surviving clades (in this case, total groups). As noted above, the crown groups, such as we simulate here, are the sum of two such processes and thus have a negative binomial distribution (see e.g. [42] for details). Based on the negative binomial distribution, 65% of all these crown groups lie between 0.5 and two times the expected clade size. Larger or smaller clades rapidly become less likely: for example, the ×10 example is, in our *homogeneous* model, extremely unlikely. However, other clades are much more likely: approximately 10% are at least two times too large; and 2% are at least three times too large. On the small side, almost 25% of clades are less than half the mean size, and 5% are less than one-fifth the mean size. We note that, although the direction of this effect is clear from our results, its precise value will depend on a set of other features. For example, the degree of influence of the clock prior versus the tree prior will be affected by the amount of useful molecular data included in the analysis. To investigate this effect, we chose one of our analyses (the 10× too large example) and re-ran it with 10, 100, 500, 1000, 2000, 5000 and 10 000 base pairs in the simulated dataset. As can be seen (figure 9), and as would be predicted, increasing the amount of data in this way (noting that all simulated base pairs mutate at precisely the clock rate provided to the model), both improves the time estimate, and also, importantly, successively better recovers the true LTT plot shape. In this case though, the data suggest that some 20 000–30 000 informative base pairs would be required to achieve a reasonable convergence to the true value, even under this ‘best case’ scenario. How real-life data with a relaxed clock compare to this performance remains to be investigated.

Most molecular clock analyses are calibrated by tip or node dating (or some combination) rather than a direct estimate of the clock rate, which will in addition typically not be appropriately modelled by a known strict rate. How such vagaries might be compensated for by accurate fossil-based dating of the earliest nodes remains to be investigated, although it appears that in general, deep node calibrations are more informative than shallow ones [106]. Clearly, if a prior range on the root (or any other node) is proposed, then the posterior result will lie within it. Nevertheless, in any model where the age of the root is conditioned on the birth–death process, the bias we demonstrate here will affect the inference of its age.

Large clades of more than two times the expected size represent some 10% of all surviving clades in our simulations. However, the Yule process, or even homogeneous birth–death process, may not necessarily reflect the true dynamics of clade diversification. If, for example, the popular diversity-dependent diversification model [69] is an intrinsic part of the diversification process, then, rather than outliers of the diversification process (albeit not particularly rare ones) such clades with their distinctive early LTT bulge would be *normal* clades, and thus, we would expect these sorts of pathologies to be very widespread. In other words, present day methods assume a *central* expectation of an exponential process with constant rate of lineage emergence, but any source of over-dispersion relative to this prior expectation (whether statistical, undersampling or ecological/evolutionary in origin) is likely to produce systematic errors of the sort we have outlined here.

As noted earlier, there are multiple ways of constructing time priors over calibrated and uncalibrated nodes, and in particular over the age of the root. These broadly separate into methods that do and do not condition the calibrated nodes on the birth–death process. The first set of these can include BEAST and BEAST2, but also more mathematically coherent methods such as the fossilized birth–death process [107]. Our results fundamentally show an influence of unrepresentative samples of the birth–death process in estimates of the root. In a package such as MCMCTree, conversely, the birth–death process does not contribute to the root prior, then, and the bias we show would not be expected to influence root or other calibrated node estimates. On the other hand, the downside to such an approach is that the time prior on calibrated nodes is sensitive to the calibration distributions and the subsequent marginal prior distributions [86,87,89], especially under relaxed clock conditions [86,87]. Thus, as has been repeatedly pointed out [89,92,93], fixing the preliminary calibration densities remains of utmost importance. Despite difficulties with assigning fossils to crown and stem groups, ascertaining the minimum constraint on calibration ranges is generally relatively straightforward, but the maximum age is much more difficult and relies on a degree of subjectivity. To take a pertinent example, [2] use the calibration points of [108], with the maximum age of crown metazoans being fixed by their absence from the Bitter Springs and Svanbergfjellet Formation biotas at *ca* 833 Ma; and the maximum age of both eumetazoans and bilaterians being fixed by their absence from the Lantian biota at *ca* 635 Ma. In addition, the presence of sponge biomarkers at *ca* 635 Ma is (under some strategies) taken as a minimum age for the origin of crown metazoans. By comparing the prior and posterior estimates of the ages of deep animal nodes in [2], one can see indeed that under a wide range of assumptions, most of the posterior estimates closely resemble the marginal priors for the deep nodes, with an exception being the bilaterians under some calibration strategies. In other words, the inferred ages of the deep nodes are being heavily influenced by calibration estimates, and rather little by the molecular clock *per se*. Ironically, then, the question of when the animals evolved still revolves around how good or bad the fossil record is considered to be. If one considers it to be bad, then deep maximum ages of deep calibration nodes will inevitably drive posterior molecular clock estimates deep too. Conversely, if one considers it to be good, then much more shallow estimates for maximum ages will be used, and this will have the opposite effect. What is clear is that palaeontologists cannot rely on molecular clocks to do their work for them in assigning affinities to problematic fossils—the posterior results are too influenced by the priors to act as a truly independent test of them. Nevertheless, we believe that the results that we present here and elsewhere [42] allow us to move beyond a simple debate about ‘evidence of absence’ versus ‘absence of evidence’. Modelling of stem and crown group dynamics combined with the order-of-appearance argument herein both strongly suggest that animals really diversified in the late Ediacaran. We therefore suggest that molecular clocks should be run with these minimum calibration priors, as presented in figure 2. In the light of this, our preferred calibration distributions would not have anywhere near as deep maximum ages as are used in [108]. It is true that there are no traces of crown-group metazoans in the Bitter Springs and Svanbergfjellet Formation biotas; but the same is surely also true of both the Lantian and the Weng’an biota. The latter in particular preserves a series of intriguing and much debated embryo-like balls of cells, but despite this demonstrating the *possibility* of crown group metazoans being preserved here [109], none have to date been found that have commanded anything close to universal assent [110]. Furthermore, although [108] regard the Lantian as providing the soft maximum for bilaterians, there is (also) surely no evidence for them in the Weng’an biota either (or, indeed, the Avalonian ‘Ediacaran’ assemblages). Thus, we would regard the age of the Weng’an biota as providing a soft maximum age for crown metazoans (especially given the increasingly problematic nature of putative sponge biomarkers (e.g. [111])) and eumetazoans; and the first appearance of bilaterian-like trace fossils as a soft maximum for crown group bilaterians. These may seem unrealistically young, but they are logically in line with the analysis we present herein. The point is thus that if the molecular data overwhelmingly refute such an age, they should be able to overcome these priors. If they are unable to do so, then the prima facie evidence of the fossil record presented herein should be retained as our best estimate of metazoan origins.

## 5. Summary

Despite its imperfections, the fossil record of early animal evolution is highly unlikely to be as grossly in error as molecular clock estimates typically indicate. In particular, neither small size nor rarity are likely to account for the purported non-appearance of crown group bilaterians in the Ediacaran or earlier. In addition, the apparent orderly appearance of taxa in the Ediacaran to Cambrian is strongly suggestive of a real-time evolutionary event being recorded. Here we have outlined a variety of survival and selection biases that can affect our understanding of major evolutionary radiations such as the Cambrian explosion, that include the disparity of the phyla. Most importantly, such biases include clade size for charismatic taxa such as the bilaterians and indeed animals as a whole; our analysis here suggests that without taking into account their unusual features, at least some types of molecular clock estimates will tend to be biased in a way that can potentially explain the mismatch between fossil and molecular clock origins of large clades. In addition, a better understanding of the dynamics of clade origins and the order of appearance of taxa in the fossil record suggests that the burden of proof for models of when the animals arose lies with those that question the basic picture that the Ediacaran to early Cambrian presents. In practical terms, this means that this record should be used as an informative prior to specify soft maxima on node ages, rather than setting them much earlier in time.

## Supplementary Material

Data and analysis code

## Data accessibility

All data and analysis code for this paper are contained in the electronic supplementary material.
